# Anti-Inflammatory Effects of the Mediterranean Diet in the Early and Late Stages of Atheroma Plaque Development

**DOI:** 10.1155/2017/3674390

**Published:** 2017-04-18

**Authors:** Rosa Casas, Mireia Urpi-Sardà, Emilio Sacanella, Sara Arranz, Dolores Corella, Olga Castañer, Rosa-María Lamuela-Raventós, Jordi Salas-Salvadó, José Lapetra, Maria P. Portillo, Ramón Estruch

**Affiliations:** ^1^Department of Internal Medicine, Hospital Clinic, Institut d'Investigació Biomèdica August Pi i Sunyer (IDIBAPS), University of Barcelona, Villarroel 170, 08036 Barcelona, Spain; ^2^Ciber Fisiopatología de la Obesidad y la Nutrición (CIBEROBN), Instituto de Salud Carlos III, Madrid, Spain; ^3^Department of Nutrition and Food Science, School of Pharmacy, University of Barcelona, Av. Joan XXIII s/n, 08028 Barcelona, Spain; ^4^Department of Epidemiology and Department of Biochemistry and Molecular Biology, School of Medicine, Valencia University, Valencia, Spain; ^5^Cardiovascular Risk and Nutrition and REGICOR Research Group, Hospital del Mar Medical Research Institute (IMIM), Barcelona, Spain; ^6^Human Nutrition Unit, Hospital Universitari de Sant Joan de Reus, IISPV, Universitat Rovira i Virgili, Reus, Spain; ^7^Department of Family Medicine, Research Unit, Distrito Sanitario Atención Primaria Sevilla, Sevilla, Spain; ^8^Nutrition and Obesity Group, Department of Nutrition and Food Sciences, Lucio Lascaray Research Center, University of Basque Country (UPV/EHU), Vitoria-Gasteiz, Spain

## Abstract

*Objective.* To evaluate the long-term effects of a Mediterranean diet (MeDiet) intervention on the plasma concentrations of inflammatory and plaque stability-related molecules in elderly people at high risk for cardiovascular disease. *Design and Setting*. 66 participants from primary care centers affiliated with the Hospital Clinic of Barcelona were randomized into 3 groups: MeDiet plus extra virgin olive oil (EVOO) or nuts and a low-fat diet (LFD). At baseline and at 3 and 5 years, we evaluated the changes in the plasma concentrations of 24 inflammatory biomarkers related to the different stages of the atherosclerotic process by Luminex®. *Results.* At 3 and 5 years, both MeDiet groups showed a significant reduction of IL-6, IL-8, MCP-1, and MIP-1*β* (*P* < 0.05; all) compared to LFD. IL-1*β*, IL-5, IL-7, IL-12p70, IL-18, TNF-*α*, IFN-*γ*, GCSF, GMCSF, and ENA78 (*P* < 0.05; all) only decreased in the MeDiet+EVOO group and E-selectin and sVCAM-1 (*P* < 0.05; both) in the MeDiet+nuts group. *Conclusions*. Long-term adherence to MeDiet decreases the plasma concentrations of inflammatory biomarkers related to different steps of atheroma plaque development in elderly persons at high cardiovascular risk.

## 1. Introduction

Atherosclerosis is a low-grade chronic inflammatory disorder of the vessel wall that involves the accumulation of lipids, especially low-density lipoproteins (LDL), in the intima. Oxidation of LDL particles (oxLDL) leads to macrophage activation and recruitment of monocytes, neutrophils, T-, and B-lymphocytes [1] in the vascular wall which induces the formation of atheroma plaque [2]. The first step of the process is the recruitment of monocytes including endothelial adhesion molecules, such as P- and E-selectins (rolling process), and subsequently, vascular cell adhesion molecule-1 (VCAM-1) and intercellular adhesion molecule-1 (ICAM-1) (migration process) [3–5]. Migration of monocytes and other immune cells is further aided by endothelial-expressed chemokines [monocyte chemotactic protein-1 (MCP-1), regulated on activation, normal T cell expressed and secreted (RANTES), macrophage inflammatory protein-1*β* (MIP-1*β*), epithelial-derived neutrophil-activating peptide 78 (ENA78), interferon-inducible T cell alpha chemoattractant (ITAC), and interferon-inducible protein- (IP-) 10] and cytokines [interleukin-6 (IL-6), IL-8, IL-12p70, IL-10, IL-18, tumor necrosis factor-*α* (TNF-*α*), interferon-*γ*-inducing factor (IFN-*γ*), etc.] [3–5]. After recruitment, monocytes in the intima differentiate into macrophages, mediated by macrophage colony-stimulating factor (M-CSF) [4–6], and thereafter release a variety of proinflammatory cytokines such as the soluble CD40 ligand, IL-1, IL-6, IL-8, IL-18, IFN-*γ*, and TNF-*α* [3–5]. The growth process of atheroma plaque leads to the formation of a fibrous cap, which contains a large number of cytokines and chemokines; some of these, such as IL-10 or IL-13, have a stability function [7] while others, such as IP-10, IL-18, and IFN-*γ*, play a role in instability [8]. Increases in some of these inflammatory biomarkers have been associated with an increased risk of cardiovascular events in patients with coronary artery disease, and thus a large number of new inflammatory biomarkers are currently being studied as possible mediators of inflammation [9].

The Mediterranean diet (MeDiet) is characterized by a high intake of extra virgin olive oil (EVOO), nuts, legumes, vegetables, fruit, fish, and whole grain products. The main nutrients of this dietary pattern are fiber, monounsaturated fatty acids (MUFA), n-3 polyunsaturated fatty acid (n-3 PUFA), vitamin C, vitamin E, and carotenoids, and all of which are associated with lower inflammation against saturated fatty acids (SFA) or trans fatty acids (TFA) and high-glucose and high-fat meals which may induce postprandial inflammation and, hence, are considered as proinflammatory factors [10]. Thus, high MUFA and n-3 PUFA intakes have been shown to exert anti-inflammatory effects. The Nurses' Health Study (80,082 healthy women between 34 and 59 years of age) showed a significant association between high intake of trans or saturated fats and an increased incidence of CVD, whereas high n-3 PUFA and MUFA intakes were associated with a decreased risk [11]. Thus, the effects of several dietary patterns such as the MeDiet, SFA or MUFA, or n-3 PUFA diets on biomarkers related to inflammation have been studied, and it has been concluded that the MeDiet exerts its anti-inflammatory and immunomodulating effect through downregulation of the expression of leukocyte adhesion molecules [12, 13], decreasing proinflammatory interleukins (IL-1, IL-6, IL-7, IL-8, and IL-18), TNF-*α* and its receptors, CRP, chemoattractant molecules (MCP-1), and soluble endothelial adhesion molecules (sVCAM-1, sICAM-1, sE-, and sP-selectins) [10–15].

However, in most of these studies, the follow-up was short, with 3 to12 weeks of intervention [16]. In addition, to date, few intervention studies have thoroughly explored the effects of diet on inflammatory biomarkers in elderly people at high cardiovascular risk. Thus, in the current study, we evaluated the long-term effects of a MeDiet intervention on most biomarkers participating in the early and late steps of atheroma plaque development in a cohort of elderly subjects enrolled in the PREDIMED study.

## 2. Methods

### 2.1. Design Overview

The PREDIMED (Prevención con DietaMediterránea) study is a 5-year, parallel-group, single-blind, multicenter, randomized, controlled feeding trial conducted in Spain which aimed to assess the effects of the MeDiet on the primary prevention of cardiovascular diseases (CVD) (http://www.predimed.es) [17, 18]. The design, methodology, and eligibility criteria for the PREDIMED study have been described previously [17, 18].

### 2.2. Setting and Participants

Recruitment took place between October 2003 and January 2009, and the 7447 participants were randomly assigned to one of the three interventions: a MeDiet supplemented with extra virgin olive oil (MeDiet+EVOO), a MeDiet supplemented with nuts (MeDiet+nuts), or a control low-fat diet (LFD). Randomization was performed centrally by means of a computer-generated random-number sequence.

The participants included were men (55 to 80 years of age) and women (60 to 80 years of age) who were free of CVD at the beginning of the study but had high cardiovascular risk because of the presence of either type 2 diabetes mellitus or at least three of the following major risk factors: current smoking, hypertension, high levels of LDL cholesterol, low levels of high-density lipoprotein cholesterol, overweight/obesity, or a family history of premature coronary heart disease (CHD). Further details of the inclusion and exclusion criteria have been published previously [17, 18]. Eligible participants were selected in primary care centers affiliated with the Hospital Clinic of Barcelona, and all the participants provided written informed consent.

In the current study, we screened 80 consecutive potential participants. Four did not fulfill the inclusion criteria, six declined to participate, and the diet could not be changed in one. After 5 years, 4 participants voluntarily left the study (1 from the MeDiet+EVOO group, 1 from the MeDiet+nuts group, and 2 from the control group). Thus, 66 were finally included in this substudy.

### 2.3. Diets and Physical Activity

All the participants were randomly assigned to one of the three intervention groups: a MeDiet+EVOO, MeDiet+nuts (walnuts, almonds, and hazelnuts), or a LFD or control diet, as described elsewhere [17, 18]. The randomized participants had an annual face-to-face interview with the dietitian. Group sessions took place every 3 months to provide the participants with descriptions of seasonal foods, shopping lists, weekly meal plans, and cooking recipes. These sessions were specific for each intervention group and included no more than 20 participants per group. In the individual sessions, a 14-item dietary screening questionnaire was used to assess adherence to the MeDiets, and a 9-item dietary screening questionnaire was used to check adherence to the control LFD [17, 18]. In addition, the individual motivational interview included a 137-item validated food frequency questionnaire (FFQ), the Minnesota leisure-time physical activity questionnaire and individualized recommendations for changes to be introduced in the participant's diet in order to achieve a personalized goal, and lastly, a 47-item questionnaire about education, lifestyle, history of illnesses, and medication use was carried out. Participants allocated to the LFD were advised to reduce all types of fat and received written recommendations according to the American Heart Association guidelines. For the 2 MeDiets, the focus was shifted to increase the intake of vegetables (≥2 servings/d), fresh fruit (≥3 servings/d), legumes, nuts, fish or seafood (≥3 servings/wk), and the use of olive oil for cooking and dressings. The detailed protocol including study design, rationale, and organization has been published elsewhere [17, 18].

Participants in the two intervention groups were given supplementary foods at no cost: either EVOO (1 liter/week for the participant and their families) or mixed nuts (30 g/day: 15 g walnuts, 7.5 g hazelnuts, and 7.5 g almonds) according to the group to which they were randomized.

Energy restriction was not specifically advised nor was physical activity promoted in any of the three groups.

### 2.4. Ethics Statement

All participants provided signed informed consent. The Institutional Review Board of the Hospital Clinic (Barcelona, Spain), accredited by the US Department of Health and Human Services (DHHS) update for Federalwide Assurance for the Protection of Human Subjects for International (Non-US) Institutions number 00000738, approved the study protocol on July 16, 2002. The trial was registered (ISRCTN35739639).

### 2.5. Laboratory Measurements

The main outcome measurements were changes in 24 plasma inflammatory biomarkers and all of which are related to the different stages of the atherosclerotic process at baseline and at 3 and 5 years of dietary intervention; IL-1*β*, IL-4, IL-5, IL-6, IL-7, IL-8, IL-10, IL-12p70, IL-13, IL-18, TNF-*α*, MCP-1, RANTES/CCL5, MIP-1*β*/CCL4, IP-10/CXCL10, IFN-*γ*, granulocyte colony-stimulating factor (GCSF), and granulocyte-macrophage colony-stimulating factor (GMCSF) were determined using the Bio-Plex Pro™ cytokine, adhesion molecule, and chemokine assays (Bio-Rad Laboratories Inc., Hercules, CA, USA) based on magnetic bead-based multiplex assays designed to measure multiple cytokines, adhesion molecules, and chemokines in plasma matrices.

On the other hand, ENA78/CXCL5, ITAC/CXCL11s, soluble VCAM-1 (sVCAM), soluble ICAM-1 (sICAM-1), and E- and P-selectins were determined using the VersaMAP™ human custom multi-analyte profiling development system (R&D Systems, Abingdon, UK) which is also based on multiplex assays designed to measure analytes in plasma matrices. Data from the reactions were acquired using the Luminex 100™ System (Luminex, Austin, TX), a high-speed digital processor that efficiently manages the data output which is further analyzed and presented as fluorescence intensity and target concentration. Thereafter, the data were processed and analyzed with the Bio-Plex Manager 6.1™ (Bio-Rad, Hercules, CA). Plasma samples were diluted 1 : 3 with the diluents provided for each assay. Concentrations were obtained by standard calibration curves. We performed all the analyses in duplicate.

### 2.6. Statistical Analyses

Statistical analyses were performed using the SPSS, version 22.0 (SPSS Inc., Chicago, IL). Variables are presented as mean and standard deviation (SD) or standard error of the mean (SEM) as appropriate. Categorical variables are expressed as percentages. Plasma inflammatory biomarkers (ITAC) had a skewed distribution (Kolmogorov and Levene tests) and were thus transformed to their natural logarithm for analysis. Changes in food and nutrient intake as well as changes in inflammatory biomarkers were measured using repeated measures ANOVA to test the effects of the interaction of 2 factors: time as a within-participants factor with 2 levels (first, at baseline and at 3 years, second, at baseline and at 5 years, and third, at 3 and 5 years) and the 3 intervention groups, adjusting for potential confounding variables such as age, sex, weight, smoking status, blood pressure systolic and diastolic, oral hypoglycemic agents, and statins and triglyceride levels. To test the effects of individual factors, we calculated the differences between 3 years and baseline and 5 years and baseline values for the adhesion molecules and inflammatory molecules and then applied an ANOVA test with the intervention group as fixed factors. Significant interactions were assessed by simple effect analysis. All the multiple contrasts were adjusted by a Bonferroni post hoc test. Within- and between-group differences were expressed as estimated means and 95% confidence interval (95% CI). Significance was set at *P* < 0.05.

## 3. Results

### 3.1. Study Population

**Figure 1:**
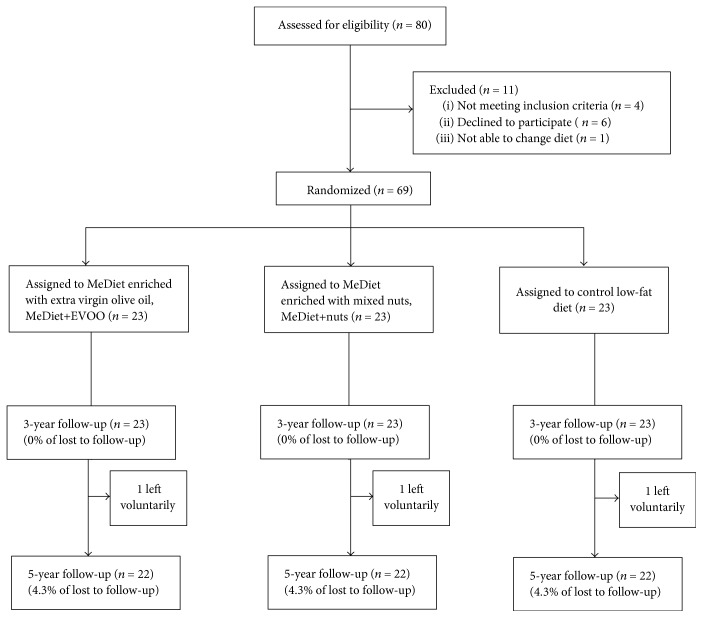
Flowchart of the study participants. The diagram includes detailed information on the participants excluded. EVOO: extra virgin olive oil; MeDiet: Mediterranean diet.

Of the 69 participants included, equal numbers (*n* = 23) were randomized into each of the three intervention groups.  Figure 1 shows the retention rates (≥95% for all) for the 3- and 5-year follow-ups. As shown in Table 1, all the participants (22 per group) selected had similar characteristics to those of the whole group (demographic characteristics, medication taken, and adiposity and CVD risk factors). We did not observe significant changes in medication use during the 5 years of intervention. On average, the participants were 67 years old and nearly half were men. Most participants (≥90%) were overweight or obese, ≥50% had hypertension and dyslipidemia, and ≥60 had diabetes.

### 3.2. Food, Energy Balance, and Dietary Adherence

As shown in Supplemental Table 1 available online at https://doi.org/10.1155/2017/3674390, we observed a significant increase in EVOO consumption (*P* = 0.001) and a decrease in refined olive oil consumption (*P* ≤ 0.003) in the MeDiet+EVOO at 3 and 5 years. Similarly, nut consumption increased in the MeDiet+nuts group (*P* ≤ 0.01) at 3 and 5 years of intervention, contrary to the other two groups. At 3 and 5 years of intervention, both MeDiets showed an increase in the consumption of vegetables (*P* ≤ 0.02), legumes (*P* ≤ 0.04), fruit (*P* < 0.05), and fish (*P* ≤ 0.02) and a reduction in the intake of cereals (*P* ≤ 0.02) and meat or meat products (*P* < 0.05). Furthermore, the consumption of cereals (*P* ≤ 0.04) and meat or meat products (*P* ≤ 0.007) decreased in the LFD group at 3 and 5 years of intervention as did the intake of vegetables at 5 years of intervention. Adherence to the MeDiet (*P* < 0.001) increased at 3 and 5 years of intervention in both MeDiet groups.

Adherence to supplemental foods was good in the two MeDiets. MUFA levels increased from baseline in the MeDiet+EVOO group (*P* < 0.001), and *α*-linolenic acid levels increased in the MeDiet+nuts group (*P* ≤ 0.009) compared to the other diets at 3 and 5 years of follow-up. A reduction in energy (*P* ≤ 0.03; both), carbohydrate (*P* ≤ 0.01; both), and cholesterol (*P* ≤ 0.03; both) intake was observed in the 3 groups at 3 and 5 years (Supplemental Table 2). Participants in the two MeDiet groups increased their intake of total fiber (*P* ≤ 0.02; both), total fat (*P* ≤ 0.04; both), and marine *ω*-3 (*P* ≤ 0.007; both) and reduced their intake of saturated fat (*P* ≤ 0.02; both). The three groups showed a reduced consumption of protein (*P* ≤ 0.04; all) after 5 years, although the control group also showed a reduction (*P* = 0.02) at 3 years of intervention. At both assessment points, polyunsaturated fatty acid (PUFA) intake significantly increased in the participants in the MeDiet+nuts group (*P* < 0.001).

### 3.3. Plasma Levels of Colony-Stimulating Factors and Soluble Endothelial Molecules

As shown in Table 2, GCSF and GMCSF (*P* ≤ 0.04, both) decreased in the MeDiet+EVOO group at 3 and 5 years of dietary intervention. The LFD group showed increased CGSF levels at 3 and 5 years (*P* ≤ 0.03). In addition, the MeDiet+nuts group showed lower serum E-selectin levels (*P* ≤ 0.02), and the LFD group showed an increase in sVCAM-1 serum levels (*P* ≤ 0.03) in both evaluations. On the other hand, the LFD group showed higher levels of P-selectin (*P* = 0.03) at 5 years of dietary intervention. No changes were observed in sICAM-1 concentrations at 3 and 5 years of intervention.

Comparisons among the 3 intervention groups showed a significant increase ≥ 40% in CGSF at 3 and 5 years in the LFD group compared to both MeDiet groups, while MeDiet+nuts participants reduced E-selectin concentrations by 14% compared to the LFD group after 5 years of intervention (*P* < 0.05; all).

### 3.4. Plasma Levels of Inflammatory Chemokines

Serum levels of MCP-1 (*P* ≤ 0.03) and MIP-1*β* (*P* < 0.05) decreased in both MeDiets at 3 and 5 years of intervention (Table 3). ENA78 and RANTES (*P* ≤ 0.02; both) decreased in the MeDiet+EVOO group at 3 and 5 years of follow-up. RANTES also decreased in the MeDiet+nuts group (*P* < 0.05) at 5 years of intervention. On the other hand, the LFD group showed an increase in RANTES (*P* ≤ 0.04), ENA78 and ITAC (*P* ≤ 0.03), and IP-10 (*P* ≤ 0.003) levels at 3 and 5 years.

Comparisons among groups showed significant reductions of 20% in MCP-1 and 15% in MIP-1*β* levels in both MeDiet groups at 3 and 5 years of intervention compared to the LFD group. On the other hand, RANTES and ENA78 increased from 25 to 50% in the LFD group compared to both MeDiet groups at 3 and 5 years of nutritional intervention.

### 3.5. Plasma Levels of Inflammatory Cytokines

After 3 and 5 years of intervention (Table 4), both MeDiet groups showed lower serum concentrations of IL-6 and IL-8 (*P* < 0.05; both) compared to baseline. Furthermore, the MeDiet+EVOO group also had lower levels of IL-1*β*, IL-5, and TNF-*α* (*P* ≤ 0.03, all), IL-7 (*P* ≤ 0.04), IL-12p70, and IFN-*γ* (*P* < 0.05; both) in both assessments. At 5 years, the MeDiet+nuts group showed an improvement in the plasma levels of IL-1*β*, IL-5, and TNF-*α* (*P* < 0.05; all), IL-7 (*P* ≤ 0.02), IL-12p70 (*P* ≤ 0.04), and IFN-*γ* (*P* ≤ 0.03). In addition, the LFD group showed an increase in the plasma concentrations of IL-7 and IL-8 (*P* < 0.05; both) at 5 years while no changes were observed in IL-4 at 3 or 5 years intervention.

On comparing the 3 intervention groups, we found significant reductions of 30–50% for IL-5, IL-12p70, TNF-*α*, and IFN-*γ*, and of 35–40% for IL-6 and IL-8 in both MeDiet groups after 5 years of intervention compared to the LFD group. Moreover, the MeDiet+EVOO group showed a significant reduction of greater than 30% in IL-1*β* after 3 and 5 years of intervention, while the LFD cohort showed an increase of 39% after 5 years of intervention compared to both MeDiet groups (*P* < 0.05; all).

### 3.6. Biomarkers Related to Plaque Stability

The MeDiet+EVOO group showed lower levels of the instability marker IL-18 (*P* ≤ 0.04) at 3 and 5 years after beginning the intervention and higher levels of the stability markers IL-13 (*P* ≤ 0.04) and IL-10 (*P* ≤ 0.03) after 5 years (Table 5). Compared to the LFD group, IL-18 levels in the MeDiet+EVOO group were reduced more than 20% after 3 and 5 years of intervention (*P* = 0.002).

## 4. Discussion

The results of the present study in elderly persons at high cardiovascular risk suggest that long-term adherence to a MeDiet could delay atheroma plaque development by reducing rolling, adhesion, and migration processes of circulatory mononuclear cells into the arterial wall. In addition, our data suggest that the MeDiet also decreases plaque vulnerability by lowering instability factors (IL-18) and increasing stability factors (IL-10 and IL-13).

Although the atherogenic process begins at very early ages (second and third decades of life), clinical events (e.g., stroke, acute myocardial infarction) develop after the age of 50 [19]. It is already known that ongoing inflammation is also crucial in the development of instability and rupture of atheromatous plaque and the subsequent appearance of ischemic events [20]. In this regard, several mechanisms have been proposed to explain how the MeDiet exerts its anti-inflammatory properties. Some evidence show that the MeDiet or some of its main foods could modulate the expression of genes related to plaque stability such as MMP-9 [21], or they may diminish the plasma levels of IL-18 [12], sSelectin-1, sICAM-1, and sVCAM-1 or other biomarkers of inflammation (TNFR-60, TNFR-80, IL-6, and CRP) [12–15, 17]. Thus, consumption of a tomato-based drink for 26 days lowered TNF-*α* secretion by 34% [22], while the consumption of EVOO and vegetables was associated with a reduction of circulating TNFR-60 [14]. In the Multi-Ethnic Study of Atherosclerosis (MESA) trial, a “vegetables and fish pattern” was inversely related to CRP and sSelectin [23] concentrations, and in a second interventional study, supplements of DHA, CRP, IL-6, GMCSF, and GCSF concentrations decreased after 90 days of intervention [24]. Several studies with olive oil, which is rich in antioxidant polyphenols, and MUFA have described reduced IL-6, sVCAM-1, and sICAM-1 levels [17, 25] in addition to other biomarkers such as CRP, IL-7, or IL-18 [26]. Finally, the intake of whole grains [23], fiber [27], or wine [28] has also been associated with an improvement in inflammatory processes reducing biomarkers such as hs-CRP, IL-6, IL-1*α*, TNF*α*-R2, MCP-1, sICAM-1, or sVCAM-1.

According to our results, long-term adherence to a MeDiet pattern seems to delay the progression of atheroma plaque and seems to agree with data published in a previous PREDIMED substudy, in which participants with a high carotid atherosclerotic burden allocated to the MeDiet supplemented with either EVOO or nuts showed reduction in the mean common carotid artery intima-media thickness (CCA-IMT) at 1 year compared with participants assigned to the control diet [29]. This healthy effect is evident in early as well as late stages of atherosclerosis since it affects biomarkers with a role in both phases of this process. Thus, monocyte/macrophage accumulation at the lesion site is a key factor in atherosclerotic disease and involves several steps including monocyte recruitment by increased levels of adhesion molecules (sVCAM, sICAM, E-, and P-selectins) and chemotactic factors (MCP-1, MIP-1*β*, IP-10, and RANTES), the induction of activation, differentiation, and proliferation processes, and immobilization of macrophages in the inflamed plaque [30]. M-CSF, which is present in the circulation, predominantly produces M2-type macrophages with increased phagocytic activity and characterized by the expression of interleukin IL-10 [30]. On the other hand, GMCSF produces M1-polarized cells with antigen presentation capacities, which express TNF-*α* and other proinflammatory cytokines such as IL-1*β*, IL-6, IL-8, and IL-12 [30]. The results of other studies [12–15, 17] agree with these results, and accordingly, both MeDiets showed an improvement in the chemokines (MCP-1, MIP-1*β*, RANTES, and ENA78), colony-stimulating (GCSF and GMCSF), cytokines (IL-1*β*, 5, 6, 7, 8, 12, TNF-*α*, and IFN-*γ*), and vulnerability plaque marker (IL-10, 13, and 18) levels.

These findings may be explained by the synergy of specific foods (fruits, vegetables, olive oil, and fish) and/or their specific nutrients such as flavonoids, *α*-tocopherol, ascorbic acid, *β*-carotene, and *ω*-3 PUFA or also by the Mediterranean pattern itself.

The suggestion that a MeDiet could modulate the expression of biomarkers implicated in the first (endothelial adhesion molecules, cytokines, chemokines, etc.) as well as the late stages (molecules related with stability of the plaque) of atheroma plaque development indicates that the MeDiet could be a good preventive tool not only in the primary but also in the secondary prevention of CVD.

The strengths of our study are its design (randomized intervention trial) excellent completion rates, close monitoring of the participants, and the myriad of inflammatory biomarkers studied. Additional strengths are the length of follow-up (5 years) and good compliance of all the participants. Nonetheless, some limitations are also acknowledged: first, the sample size was relatively small and second, the results cannot be generalized to the overall population because our participants were older subjects at high risk for CVD living in a Mediterranean country.

## 5. Conclusions

The present study supports the recommendation that the MeDiet is a very useful dietary pattern in the primary prevention of CVD. The results of this study show that the MeDiet and its antioxidant compounds interfere with the atherosclerotic inflammatory process by downregulating proinflammatory biomarkers and upregulating biomarkers related to atheroma stability plaque. Moreover, our results suggest that this anti-inflammatory effect is maintained in the long term.

## Supplementary Material

Supplemental Table 1. Baseline values and changes in consumption of key food items, 14-point Mediterranean diet score and physical activity at 3 and 5 years of follow-up after a MeDiet+EVOO, MeDiet+Nuts or LFD intervention in subjects at high risk for cardiovascular disease. Supplementary Table 2. Changes in baseline energy and nutrient intake.

## Figures and Tables

**Table 1:** Baseline characteristics of the participants at high risk for cardiovascular disease included in the study and classified according to the dietary intervention administered.

	MeDiet+EVOO	MeDiet+nuts	Low-fat diet	*P* ^2^
Age, years	67.8 ± 4.8^1^	66.0 ± 5.8	66.0 ± 7.1	0.50
Men, *n* (%)	10 (46)^1^	12 (55)	9 (41)	0.66
Family history of CHD, *n* (%)	5 (23)	4 (18)	7 (32)	0.56
Smoking status, *n* (%)				0.33
Never smoked	17 (72)	12 (55)	14 (64)	
Former smoker	4 (18)	6 (27)	3 (14)	
Current smoker	1 (4)	4 (18)	5 (22)	
BMI, kg/m^2^	29.7 ± 3.7	30.4 ± 3.2	28.5 ± 3.6	0.20
BMI ≥ 25 kg/m^2^, *n* (%)	22 (100)	21 (96)	20 (90)	0.29
Waist circumference, cm	101 ± 10	106 ± 7	102 ± 7	0.11
Type 2 diabetes, *n* (%)	16 (73)	15 (68)	13 (59)	0.62
Hypertension, *n* (%)	18 (82)	18 (82)	14 (64)	0.27
Dyslipidemia, *n* (%)	10 (46)	13 (64)	13 (64)	0.58
Medications, *n* (%)				
ACE inhibitors	2 (9)	3 (14)	5 (23)	0.44
Diuretics	4 (18)	5 (23)	4 (18)	0.91
Other antihypertensive agents	1 (5)	2 (9)	1 (5)	0.77
Statins	4 (18)	5 (23)	4 (18)	0.91
Other lipid-lowering agents	1 (5)	0 (0)	2 (9)	0.35
Insulin	1 (5)	3 (14)	3 (14)	0.53
Oral hypoglycemic drugs	9 (41)	8 (36)	9 (41)	0.94
Aspirin or antiplatelet drugs	1 (5)	6 (27)	3 (14)	0.11
NSAIDS	3 (14)	6 (27)	3 (14)	0.40

^1^Values are means ± SDs, *n* = 22 unless expressed otherwise. ^2^From Pearson's chi-square test for categorical variables and one-factor ANOVA for continuous variables. ACE: angiotensin-converting enzyme; BMI: body mass index; CHD: coronary heart disease; EVOO: extra virgin olive oil; MeDiet+EVOO: Mediterranean diet supplemented with extra virgin olive oil; MeDiet+nuts: Mediterranean diet supplemented with nuts; NSAIDS: nonsteroidal anti-inflammatory drugs.

**Table 2:** Baseline values and changes in the plasma concentrations of colony-stimulating factors and soluble adhesion molecules at 3 and 5 years of follow-up after a MeDiet+EVOO, MeDiet+nuts, or LFD intervention in subjects at high risk for cardiovascular disease.

		Intervention group			Between-group changes *P* value for differences^3^		
		MeDiet+EVOO	MeDiet+nuts	LFD	MeDiet+EVOO versus LFD	MeDiet+EVOO versus MeDiet+nuts	MeDiet+nuts versus LFD
GCSF, pg/mL	Baseline^1^	8.7 ± 7.5	7.6 ± 6.0	6.0 ± 5.8			
	3 y^2^	−3.6 (−6.2, −1.0)^∗^	−2.0 (−4.5, 0.5)	2.9 (0.3, 5.4)^∗^	0.002	1.00	0.008
	5 y^2^	−3.8 (−6.4, −1.1)^∗^	−0.9 (−3.5, 1.8)	3.8 (1.0, 6.5)^∗^	0.001	0.44	0.006

GMCSF, pg/mL	Baseline	3.5 ± 3.0	2.7 ± 2.6	2.3 ± 1.4			
	3 y	−1.7 (−2.8, −0.6)^∗^	−0.08 (−1.1, 0.9)	0.5 (−0.7, 1.7)	0.08	0.27	1.00
	5 y	−1.6 (−3.0, −0.1)^∗^	−1.1 (−2.5, 0.2)	0.5 (−1.0, 2.0)	0.09	1.00	0.18

E-selectin, pg/mL	Baseline	7314 ± 3898	9331 ± 3675	8174 ± 4828			
	3 y	−56 (−1179, 1066)	−1563 (−2798, −329)^∗^	−468 (−1601, 665)	1.00	0.13	0.42
	5 y	298 (−953, 1549)	−3054 (−4230, −1878)^∗^^,*γ*^	−372 (−1638, 894)	1.00	0.001	0.008

P-selectin, pg/mL	Baseline	9355 ± 4531	11,262 ± 4884	9372 ± 4521			
	3 y	−841 (−2139, 456)	−626 (−1843, 591)	403 (−835, 1641)	0.40	1.00	1.00
	5 y	−486 (−2389, 1417)	555 (−1295, 2406)	1980 (130, 3829)^∗^^,*γ*^	0.42	1.00	0.63

sVCAM-1, ng/mL	Baseline	281 ± 168	363 ± 267	229 ± 168			
	3 y	−19.2 (−79.6, 41.2)	−13.9 (−73.4, 45.6)	−81.1 (20.5, 142)^∗^	0.11	1.00	0.13
	5 y	−14.5 (−73.4, 44.4)	3.2 (−54.0, 60.4)	67.0 (7.0, 127)^∗^	0.46	1.00	0.57

^1^Values are means ± SDs, *n* = 22 unless expressed otherwise. 2Mean differences (95% CI). ^∗^*P*: different from baseline, (*P* < 0.05). *^γ^P*: different from 3 and 5 y of intervention (*P* < 0.05). ^3^*P* value: significant differences (*P* < 0.05) in changes between groups. EVOO: extra virgin olive oil; LFD: low-fat diet; MeDiet+EVOO: Mediterranean diet supplemented with extra virgin olive oil; MeDiet+nuts: Mediterranean diet supplemented with nuts.

**Table 3:** Baseline values and changes in the plasma concentrations of chemokines at 3 and 5 years of follow-up after a MeDiet+EVOO, MeDiet+nuts, or LFD intervention in subjects at high risk for cardiovascular disease.

		Intervention group			Between-group changes *P* value for differences^3^		
		MeDiet+EVOO	MeDiet+nuts	LFD	MeDiet+EVOO versus LFD	MeDiet+EVOO versus MeDiet+nuts	MeDiet+nuts versus LFD
MCP-1, pg/mL	Baseline^1^	4.3 ± 2.4	4.2 ± 2.3	3.1 ± 1.4			
	3 y^2^	−1.1 (−1.7, −0.4)^∗^	−0.7 (−1.4, −0.1)^∗^	0.4 (−0.3, 1.0)	0.01	0.84	0.16
	5 y^2^	−0.9 (−1.8, −0.1)^∗^	−1.2 (−2.1, −0.4)^∗^	0.8 (−0.08, 1.6)	0.02	1.00	0.01

MIP-1*β*, pg/mL	Baseline	9.5 ± 4.1	8.8 ± 5.8	7.7 ± 3.3			
	3 y	−1.1 (−2.2, −0.03)^∗^	−1.1 (−2.2, −0.09)^∗^	0.6 (−0.5, 1.7)	0.02	1.00	0.02
	5 y	−1.8 (−3.2, −0.4)^∗^	−1.4 (−2.8, −0.06)^∗^	0.60 (−0.8, 2.0)	0.02	1.00	0.04

RANTES, pg/mL	Baseline	1040 ± 317	952 ± 439	901 ± 355			
	3 y	−250 (−416, −85)^∗^	−89 (−249, 71)	199 (35, 362)^∗^	<0.001	0.50	0.02
	5 y	−276 (−509, −44)^∗^^,a^	−230 (−454, −5.2)^∗^^,a^	242 (13, 471)^∗^	0.001	1.00	0.003

ENA78, pg/mL	Baseline	185 ± 93	168 ± 123	161 ± 135			
	3 y	−68 (−122, −14)^∗^^,a^	−13 (−62, 37)	56 (5.7, 106)^∗^	0.004	0.34	0.22
	5 y	−101 (−174, −27)^∗^^,a^	−32 (−102, 39)	88 (17, 159)^∗^	0.001	0.60	0.03

ITAC, pg/mL	Baseline	4.3 ± 3.0	4.4 ± 3.0	3.0 ± 2.0			
	3 y	1.2 (−1.1, 3.6)	1.5 (−0.8, 3.7)	1.9 (0.3, 3.9)^∗^	0.74	1.00	0.82
	5 y	0.7 (−3.0, 4.4)	3.2 (−0.4, 6.8)	4.8 (1.3, 8.3)^∗^	1.00	1.00	1.00

IP-10, pg/mL	Baseline	21.3 ± 9.4	25.2 ± 13.7	15.4 ± 10.1			
	3 y	4.6 (−2.0, 11.1)	0.9 (−5.8, 7.5)	10.5 (3.8, 17.3)^∗^	0.27	1.00	0.18
	5 y	3.3 (−7.7, 14.4)	0.8 (−10.2, 11.7)	18.1 (6.8, 29.4)^∗^	0.25	1.00	0.23

^1^Values are means ± SDs, *n* = 22 unless expressed otherwise. ^2^Mean differences (95% CI). ^∗^*P*: different from baseline, (*P* < 0.05). ^3^*P* value: significant differences (*P* < 0.05) in changes between groups. ^a^MeDiet+EVOO versus MeDiet+nuts are significantly different, *P* < 0.05. EVOO: extra virgin olive oil; LFD: low-fat diet; MeDiet+EVOO: Mediterranean diet supplemented with extra virgin olive oil; MeDiet+nuts: Mediterranean diet supplemented with nuts.

**Table 4:** Baseline values and changes in the plasma concentrations of cytokines at 3 and 5 years of follow-up after a MeDiet+EVOO, MeDiet+nuts, or LFD intervention in subjects at high risk for cardiovascular disease.

		Intervention group			Between-group changes *P* value for differences^3^		
		MeDiet+EVOO	MeDiet+nuts	LFD	MeDiet+EVOO versus LFD	MeDiet+EVOO versus MeDiet+nuts	MeDiet+nuts versus LFD
IL-1*β*, pg/mL	Baseline^1^	0.23 ± 0.23	0.16 ± 0.13	0.17 ± 0.15			
	3 y^2^	−0.10 (−0.18, −0.02)^∗^	−0.01 (−0.09, 0.07)	0.06 (−0.02, 0.14)	0.01	0.29	0.59
	5 y^2^	−0.08 (−0.16, −0.01)^∗^	−0.07 (−0.14, −0.02)^∗^^,*γ*^	0.03 (−0.05, 0.1)	0.02	1.00	0.06

IL-5, pg/mL	Baseline	0.51 ± 0.52	0.43 ± 0.39	0.41 ± 0.37			
	3 y	−0.22 (−0.38, −0.05)^∗^	−0.04 (−0.20, 0.13)	0.12 (−0.04, 0.29)	0.04	0.40	0.32
	5 y	−0.25 (−0.43, −0.07)^∗^^,a^	−0.17 (−0.33, −0.02)^∗^^,*γ*^	0.14 (−0.01, 0.31)	0.03	1.00	0.02

IL-6, pg/mL	Baseline	1.16 ± 1.20	1.22 ± 0.92	0.79 ± 0.66			
	3 y	−0.40 (−0.79, −0.01)^∗^	−0.42 (−0.81, −0.04)^∗^	0.18 (−0.21, 0.57)	0.06	1.00	0.06
	5 y	−0.45 (−0.90, −0.03)^∗^	−0.57 (−1.00, −0.13)^∗^	0.26 (−0.20, 0.71)	0.94	1.00	0.02

IL-7, pg/mL	Baseline	0.97 ± 0.77	0.88 ± 0.59	0.66 ± 0.46			
	3 y	−0.32 (−0.63, −0.01)^∗^	−0.12 (−0.41, 0.17)	0.18 (−0.12, 0.47)	0.02	0.97	0.23
	5 y	−0.44 (−0.77, −0.12)^∗^	−0.36 (−0.66, −0.05)^∗^^,*γ*^	0.31 (0.01, 0.62)^∗^	0.001	1.00	0.001

IL-8, pg/mL	Baseline	2.39 ± 2.04	2.18 ± 1.29	1.85 ± 1.42			
	3 y	−0.73 (−1.38, −0.07)^∗^	−0.66 (−1.31, −0.02)^∗^	0.53 (−0.15, 1.20)	0.02	1.00	0.04
	5 y	−0.88 (−1.60, −0.19)^∗^^,a^	−0.82 (−1.51, −0.12)^∗^^,a^	0.73 (0.02, 1.45)^∗^	0.006	1.00	0.01

IL-12p70, pg/mL	Baseline	2.02 ± 1.94	1.42 ± 1.03	1.58 ± 2.7			
	3 y	−0.85 (−1.69, −0.01)^∗^	−0.32 (−1.15, 0.51)	−0.31 (−1.13, 0.51)	0.12	1.00	1.00
	5 y	−0.78 (−1.55, −0.01)^∗^	−0.80 (−1.54, −0.06)^∗^	−0.29 (−1.05, 0.47)	0.37	1.00	0.39

TNF-*α*, pg/mL	Baseline	2.91 ± 2.91	2.47 ± 2.29	1.80 ± 1.76			
	3 y	−1.42 (−2.57, −0.27)^∗^	−0.44 (−1.49, 0.61)	0.59 (−0.48, 1.66)	0.01	0.56	0.31
	5 y	−1.57 (−2.80, −0.33)^∗^	−1.16 (−2.32, −0.01)^∗^	0.61 (−0.58, 1.80)	0.03	1.00	0.77

IFN-*γ*, pg/mL	Baseline	14.4 ± 9.1	12.7 ± 11.1	9.0 ± 6.9			
	3 y	−4.1 (−8.3, −0.01)^∗^	−1.4 (−5.4, 2.7)	2.1 (−2.0, 6.3)	0.05	0.74	0.60
	5 y	−6.2 (−11.1, −1.3)^∗^	−5.3 (−10.0, −0.6)^∗^	3.2 (−1.7, 8.1)	0.02	1.00	0.03

^1^Values are means ± SDs, *n* = 22 unless expressed otherwise. ^2^Mean differences (95% CI). ^∗^*P*: different from baseline, (*P* < 0.05). *^γ^P*: different from 3 and 5 y of intervention (*P* < 0.05). ^3^*P* value: significant differences (*P* < 0.05) in changes between groups. ^a^MeDiet+EVOO versus MeDiet+nuts are significantly different, *P* < 0.05. EVOO: extra virgin olive oil; LFD: low-fat diet; MeDiet+EVOO: Mediterranean diet supplemented with extra virgin olive oil; MeDiet+nuts: Mediterranean diet supplemented with nuts.

**Table 5:** Baseline values and changes in the plasma concentrations of molecules related to the vulnerability of atheroma plaque formation at 3 and 5 years of follow-up after a MeDiet+EVOO, MeDiet+nuts, or LFD intervention in subjects at high risk for cardiovascular disease.

		Intervention group			Between-group changes *P* value for differences^3^		
		MeDiet+EVOO	MeDiet+nuts	LFD	MeDiet+EVOO versus LFD	MeDiet+EVOO versus MeDiet+nuts	MeDiet+nuts versus LFD
IL-10, pg/mL	Baseline^1^	0.38 ± 0.37	0.36 ± 0.34	0.32 ± 0.38			
	3 y^2^	0.01 (−0.14, 0.16)	0.06 (−0.09, 0.21)	0.01 (−0.16, 0.16)	1.00	1.00	1.00
	5 y^2^	0.34 (0.02, 0.66)^∗^^,*γ*^	−0.003 (−0.31, 0.30)	−0.08 (−0.41, 0.25)	0.29	0.63	1.00

IL-13, pg/mL	Baseline	0.25 ± 0.52	0.20 ± 0.39	0.34 ± 0.37			
	3 y	−0.02 (−0.21, 0.18)	0.07 (−0.11, 0.25)	0.11 (−0.08, 0.31)	1.00	1.00	1.00
	5 y	0.26 (0.02, 0.49)^∗^^,*γ*^	0.07 (−0.16, 0.03)	−0.14 (−0.37, 0.09)	0.05	1.00	0.36

IL-18, pg/mL	Baseline	8.7 ± 5.7	10.7 ± 5.2	7.5 ± 3.3			
	3 y	−1.8 (−3.6, −0.07)^∗^^,a^	−0.6 (−2.3, 1.2)	1.1 (−0.6, 2.9)	0.02	0.21	1.00
	5y	−1.9 (−0.5, 3.0)^∗^^,a^	1.1 (−0.7, 2.9)	1.3 (−0.5, 3.0)	0.02	0.02	1.00

^1^Values are means ± SDs, *n* = 22 unless expressed otherwise. ^2^Mean differences (95% CI). ^∗^*P*: different from baseline, (*P* < 0.05). *^γ^P*: different from 3 and 5 y of intervention (*P* < 0.05). ^3^*P* value: significant differences (*P* < 0.05) in changes between groups. ^a^MeDiet+EVOO or MeDiet+nuts versus low-fat diet are significantly different, *P* < 0.05. EVOO: extra virgin olive oil; LFD: low-fat diet; MeDiet+EVOO: Mediterranean diet supplemented with extra virgin olive oil; MeDiet+nuts: Mediterranean diet supplemented with nuts.
