# Supplementary material for: Anti-Inflammatory Effects of the Mediterranean Diet in the Early and Late Stages of Atheroma Plaque Development
Source: Mediators Inflamm. 2017 Apr 18;2017:3674390. doi: 10.1155/2017/3674390 (PMC5412172; doi:10.1155/2017/3674390)
Supplement: Supplementary file 1 — Supplemental Table 1. Baseline values and changes in consumption of key food items, 14-point Mediterranean diet score and physical activity at 3 and 5 years of follow-up after a MeDiet+EVOO, MeDiet+Nuts or LFD intervention in subjects at high risk for cardiovascular disease. Supplementary Table 2. Changes in baseline energy and nutrient intake. [file 3674390.f1.docx]

**Supplemental TABLE 1. Baseline values and changes in consumption of key food items, 14-point Mediterranean diet score and physical activity at 3 and 5 years of follow-up after a MeDiet+EVOO, MeDiet+Nuts or LFD intervention in subjects at high risk for cardiovascular disease.**

|  |  |  | Intervention Group |  |  |
| --- | --- | --- | --- | --- | --- |
|  |  | MeDiet + EVOO | MeDiet + Nuts | Low-fat diet | *Time x treatment ^3^* |
| EVOO, *g/d* | Baseline^1^ | 24.7 ± 22.7 | 18.0 ± 17.2 | 22.3 ± 16.5 |  |
|  | 3y.^2^ | 20.9 (9.3, 32.5)*^,a,b^ | -0.8 (-12.3, 10.8) | -4.5 (-16.1, 7.1) | <0.001 |
|  | 5y.^2^ | 20.7 (9.1, 32.3)*^,a,b^ | 0.8 (-10.8, 12.4) | -3.0 (-14.5, 8.7) | 0.001 |
| Refined OO,  *g/d* | Baseline | 22.7 ± 14.7 | 19.9 ± 18.2 | 25.0 ± 14.8 |  |
|  | 3y. | -14.3 (-23.5, -5.0)*^,a,b^ | 4.0 (-5.3, 13.2) | 1.6 (-7.6, 10.9) | 0.07 |
|  | 5y. | -17.7 (-26.9, -8.5)*^,a,b^ | 0.6 (-8.6, 9.7) | -0.6 (-9.8, 8.5) | 0.03 |
| Total nuts,  *g/d* | Baseline | 14.6 ± 13.3 | 13.4 ± 19.6 | 8.7 ± 10.1 |  |
|  | 3y. | -5.6 (-15.2, 3.9)^b^ | 13.5 (4.0, 23.1)*^,a^ | 4.2 (-5.4, 13.8) | 0.04 |
|  | 5y. | -9.0 (-19.3, 1.4) ^b^ | 13.6 (3.3, 24.0)*^,a^ | 2.4 (-8.0, 12.8) | 0.01 |
| Vegetables,  *g/d* | Baseline | 419 ± 146 | 330 ± 133 | 379 ± 132 |  |
|  | 3y. | 144 (65, 223)*^,γ,a^ | 101 (23, 180)* | 20 (-59, 99) | 0.01 |
|  | 5y. | 75 (13, 138)*^,a^ | 103 (40, 165)* | -36 (-100, 27) | 0.03 |
| Legumes,  *g/d* | Baseline | 18.1 ± 5.5 | 21.1 ± 6.8 | 19.3 ± 11.1 |  |
|  | 3y. | 6.7 (0.5, 13.0)* | 6.6 (0.4, 12.8)* | 5.1 (-1.1, 11.4) | 0.33 |
|  | 5y. | 7.0 (1.2, 12.8)* | 6.3 (0.5, 12.1)* | 3.2 (-2.6, 9.0) | 0.29 |
| Fruits, *g/d* | Baseline | 494 ± 227 | 372 ± 187 | 488 ± 223 |  |
|  | 3y. | 140 (32, 247)* | 180 (73, 287)* | 49 (-59, 156) | 0.18 |
|  | 5y. | 109 (1, 217)*^,a^ | 135 (27, 243)* | -90 (-198, 18)^γ^ | 0.09 |
| Cereals,  *g/d* | Baseline | 274 ± 98 | 260 ± 107 | 275 ± 115 |  |
|  | 3y. | -78 (-123, -33)* | -53 (-98, -9)* | -59 (-104, -14)* | 0.85 |
|  | 5y. | -98 (-143, -52)* | -89 (-134, -43)*^,γ^ | -92 (-138, -47)*^,γ^ | 0.81 |
| Fish or seafood,  *g/d* | Baseline | 93.8 ± 44.1 | 94.9 ± 44.2 | 114 ± 32.7 |  |
|  | 3y. | 23.8 (7.3, 40.4)* | 20.2 (3.6, 36.7)* | 10.8 (-5.7, 27.3) | 0.33 |
|  | 5y. | 30.7 (13.2, 48.2)* | 45.0 (27.4, 62.5)*^,γ^ | 2.7 (-14.9, 20.2) | 0.78 |
| Meat or meat products,  *g/d* | Baseline | 141 ± 67 | 172 ± 52 | 159 ± 51 |  |
|  | 3y. | -35.4 (-60.8, -9.9)*^,b^ | -26.5 (-52.5, -0.4)* | -31.7 (-57.2, -6.2)* | 0.04 |
|  | 5y. | -31.9 (-56.1, -7.6)* | -45.3 (-69.0, -21.6)*^,γ^ | -40.4 (-64.1, -16.7)* | 0.32 |
| Pastries, cakes or sweets,  *g/d* | Baseline | 20.3 ± 18.0 | 22.1 ± 25.7 | 23.0 ± 60.8 |  |
|  | 3y. | -5.7 (-19.9, 8.5) | -4.2 (-18.4, 10.0) | -8.0 (-22.2, 6.2) | 0.93 |
|  | 5y. | -3.6 (-21.5, 14.2) | -5.8 (-23.6, 12.0) | -12.3 (-30.2, 5.5) | 0.94 |
| Dairy products,  *g/d* | Baseline | 374 ± 128 | 340 ± 196 | 372 ± 271 |  |
|  | 3y. | 17.1 (-100, 135) | -25.5 (-140, 89.2) | 1.6 (-116, 119) | 0.57 |
|  | 5y. | -50.2 (-144, 43.3) | -32.1 (-145, 81.0) | -25.6 (-128, 76.3) | 0.72 |
| Alcohol,  *g/d* | Baseline | 10.7 ± 12.9 | 6.5 ± 6.4 | 7.5 ± 11.0 |  |
|  | 3y. | 1.1 (-1.2, 3.5) | 1.2 (-1.2, 3.5) | 0.7 (-1.6, 3.1) | 0.36 |
|  | 5y. | 2.3 (-0.6, 5.2) | 1.3 (-1.6, 4.2) | -0.4 (-3.3, 2.4) | 0.27 |
| Wine, *mL/d* | Baseline | 30.0 ± 61.4 | 25.0 ± 29.7 | 35.1 ± 55.7 |  |
|  | 3y. | 11.6 (-8.1, 31.2) | 7.1 (-12.6, 26.8) | 4.7 (-15.5, 24.8) | 0.80 |
|  | 5y. | 5.1 (-15.2, 25.4) | 11.0 (-9.4, 31.3) | -5.2 (-25.6, 15.1) | 0.99 |
| Physical Activity, *Kcal/d* | Baseline | 317 ± 205 | 236 ± 267 | 282 ± 215 |  |
|  | 3y. | 8.7 (-89.6, 107) | 4.5 (-93.8, 103) | 43.7 (-54.6, 142) | 0.34 |
|  | 5y. | 11.7 (-81.4, 105) | 12.8 (-80.2, 106) | -9.7 (-103, 83.3) | 0.40 |
| MeDiet Score | Baseline | 9.0 ± 1.5 | 8.0 ± 1.9 | 8.1 ± 1.4 |  |
|  | 3y. | 1.7 (1.3, 2.2)*^,a^ | 1.5 (1.1, 1.9)*^,a^ | 0.05 (-0.4, 0.5) | 0.002 |
|  | 5y. | 1.5 (1.0, 2.1)*^,a^ | 1.7 (1.2, 2.2)*^,a^ | 0.1 (-0.4, 0.7) | 0.003 |

^1^Values are means ± SDs, n=54 or 52 (LFD) unless expressed otherwise.

^2^Mean differences (95% CI). ^*^*P*: Different from baseline, (*P<*0.05). ^γ^*P*: Different from 3 and 5y of intervention (*P<*0.05).

^3^Time x treatment: comparison between measures obtained before and after intervention and among the 3 diet groups, *P<*0.05. ^a^MeDiet+EVOO or MeDiet+nuts vs. low fat-diet and ^b^MeDiet+EVOO vs. MeDiet+nuts are significantly different, *P<*0.05**.** LFD, low-fat diet; MeDiet+EVOO, Mediterranean diet supplemented with extra virgin olive oil; MeDiet+Nuts, Mediterranean diet supplemented with nuts.

**Supplementary TABLE 2.** Changes in baseline energy and nutrient intake.

|  |  | Within-group mean changes | | | Between-group changes^4^ | | | |
| --- | --- | --- | --- | --- | --- | --- | --- | --- |
|  |  | MeDiet + EVOO (n=22) | MeDiet + Nuts (n=22) | Low-fat diet (n=22) |  | MeDiet+EVOO vs. LFD | MeDiet+EVOO vs. MeDiet+Nuts | MeDiet+Nuts vs. LFD |
|  |  | Mean | Mean | Mean | *Pint^3^* | *P* | *P* | *P* |
| Energy (kcal/d) | Baseline^1^ | 2484 ± 529 | 2609 ± 591 | 2430 ± 794 |  |  |  |  |
|  | 3y.^2^ | -246 (-466, -25.9)* | -241 (-456, -26.6)* | -257 (-477, -36.7)* | 0.42 | 1.00 | 1.00 | 1.00 |
|  | 5y.^2^ | -552 (-795, -309)* | -392 (-685, -99.0)* | -612 (-877, -348)* | 0.42 | 1.00 | 1.00 | 0.80 |
| Protein (g) | Baseline | 101 ± 21 | 107 ± 23 | 102 ± 23 |  |  |  |  |
|  | 3y. | -8.9 (-18.8, 1.1) | -5.4 (-15.1, 4.4) | -12.5 (-22.5, -2.5)* | 0.20 | 1.00 | 1.00 | 0.93 |
|  | 5y. | -19.9 (-29.5, -10.3)* | -12.6 (-24.2, -0.9)* | -19.1 (-29.6, -8.6)* | 0.53 | 1.00 | 1.00 | 1.00 |
| Carbohydrate (g) | Baseline | 267 ± 65 | 275 ± 92 | 270 ± 125 |  |  |  |  |
|  | 3y. | -45.2 (-80.6, -9.8)* | -55.0 (-90.0, -20.4)* | -53.3 (-88.7, -18.0)* | 0.98 | 1.00 | 1.00 | 1.00 |
|  | 5y. | -83.2 (-124, -42.9)* | -93.9 (-143, -45.2)* | -108 (-152, -63.8)* | 0.79 | 1.00 | 1.00 | 1.00 |
| Fiber (g/d) | Baseline | 28.2 ± 4.1 | 27.0 ± 7.3 | 26.5 ± 6.3 |  |  |  |  |
|  | 3y. | 3.4 (0.5, 6.2)* | 4.6 (1.8, 7.5)* | 0.3 (-2.5, 3.2) | 0.17 | 0.42 | 1.00 | 0.12 |
|  | 5y. | 3.0 (0.8, 5.3)* | 3.5 (1.2, 5.8)*^,a^ | -0.6 (-3.0, 1.7) | 0.15 | 0.80 | 1.00 | 0.04 |
| Total fat (g) | Baseline | 101 ± 24.5 | 104 ± 21.0 | 97.3 ± 31.7 |  |  |  |  |
|  | 3y. | 13.0 (1.6, 24.4)* | 17.3 (6.0, 28.7)*^,b^ | 2.6 (-8.8, 14.0) | 0.06 | 0.61 | 1.00 | 0.22 |
|  | 5y. | 8.9 (0.03, 17.8)* | 11.2 (2.3, 20.1)* | 1.2 (-7.7, 10.1) | 0.27 | 0.68 | 1.00 | 0.35 |
| SFA (g) | Baseline | 29.8 ± 9.5 | 30.1 ± 8.3 | 28.3 ± 7.2 |  |  |  |  |
|  | 3y. | -5.3 (-8.4, -2.2)* | -3.7 (-6.8, -0.6)* | -1.8 (-5.0, 1.3) | 0.82 | 0.37 | 1.00 | 1.00 |
|  | 5y. | -6.6 (-10.2, -3.0)* | -5.7 (-9.3, -2.1)* | -2.4 (-6.0, 1.2) | 0.90 | 0.32 | 1.00 | 0.63 |
| MUFA (g) | Baseline | 46.3 ± 15.0 | 51.6 ± 13.9 | 51.3 ± 11.8 |  |  |  |  |
|  | 3y. | 11.6 (5.7, 17.4)*^,a^ | 1.5 (-4.3, 7.3) | 0.06 (-5.8, 5.9) | 0.95 | 0.02 | 0.04 | 1.00 |
|  | 5y. | 11.0 (5.4, 16.5)*^,a^ | -1.7 (-7.2, 3.9) | -5.2 (-10.7, 0.4) | 0.68 | <0.001 | 0.006 | 1.00 |
| PUFA (g) | Baseline | 16.7 ± 5.4 | 16.5 ± 5.5 | 15.9 ± 4.6 |  |  |  |  |
|  | 3y. | -0.5 (-3.4, 2.4) ^b^ | 5.8 (2.9, 8.7)* | 2.2 (-0.7, 5.1) | 0.09 | 0.60 | 0.01 | 0.25 |
|  | 5y. | -2.5 (-5.1, 0.2) ^b^ | 4.4 (1.8, 7.0)* | 0.9 (-1.8, 3.5) | 0.08 | 0.23 | 0.001 | 0.19 |
| Linoleic acid, (g/d) | Baseline | 12.8 ± 3.8 | 14.1 ± 5.9 | 13.4 ± 7.3 |  |  |  |  |
|  | 3y. | -0.3 (-2.8, 2.2)^b^ | 1.9 (-0.6, 4.5) ^a^ | -1.7 (-4.3, 0.8) | 0.04 | 1.00 | 0.68 | 0.14 |
|  | 5y. | -2.1 (-4.6, 0.4) ^b^ | 0.4 (-2.1, 2.9) ^a^ | -2.1 (-4.6, 0.4) | 0.06 | 1.00 | 0.50 | 0.46 |
| α-linolenic acid , (g/d) | Baseline | 1.8 ± 0.7 | 1.8 ± 0.7 | 1.7 ± 0.8 |  |  |  |  |
|  | 3y. | -0.3 (-0.7, 0.2)^b^ | 0.7 (0.2, 1.1)*^, a^ | -0.2 (-0.7, 0.2) | 0.002 | 1.00 | 0.009 | 0.01 |
|  | 5y. | -0.3 (-0.8, 0.2)^b^ | 0.8 (0.3, 1.2)*^, a^ | -0.1 (-0.6, 0.4) | 0.007 | 1.00 | 0.007 | 0.04 |
| Marine n-3 fatty acids, (g/d) | Baseline | 0.8 ± 0.5 | 0.9 ± 0.5 | 0.7 ± 0.4 |  |  |  |  |
|  | 3y. | 0.2 (0.06, 0.4)* | 0.4 (0.2, 0.6)*^,a^ | 0.05 (-0.1, 0.2) | 0.02 | 0.40 | 0.35 | 0.008 |
|  | 5y. | 0.3 (0.1, 0.4)* | 0.4 (0.3, 0.6)* | 0.2 (-0.002, 0.3) | 0.16 | 1.00 | 0.50 | 0.09 |
| Cholesterol, (mg/d) | Baseline | 452 ± 128 | 439 ± 117 | 421 ± 130 |  |  |  |  |
|  | 3y. | -62 (-111, -14)* | -55 (-103, -6)* | -71 (-119, -22)* | 0.46 | 1.00 | 1.00 | 1.00 |
|  | 5y. | -94 (-141, -48)* | -91 (-137, -44)* | -96 (-142, -49)* | 0.54 | 1.00 | 1.00 | 1.00 |

Data analyzed by repeated-measures 2-factor ANOVA (simple-effect analysis by Bonferroni’s multiple contrast).^1^Values are mean ± SD. ^2^Mean differences (95% CI). ^*^P: Significant differences (P<0.05) between before and after the intervention. ^3^Pint: comparison between measures obtained before and after intervention and among the 3 diet groups, P<0.05. ^4^Pvalue: Significant differences (P<0.05) between-group changes (Data analyzed by ANCOVA test, with the intervention group as fixed factor). ^a^MeDiet+EVOO or MeDiet+nuts vs. low fat-diet and ^b^MeDiet+EVOO vs. MeDiet+nuts are significantly different, P<0.05. EVOO, extra virgin olive oil; MeDiet+EVOO, Mediterranean diet supplemented with extra virgin olive oil; MeDiet+Nuts, Mediterranean diet supplemented with nuts. EVOO, extra virgin olive oil; MeDiet+EVOO, Mediterranean diet supplemented with extra virgin olive oil; MeDiet+Nuts, Mediterranean diet supplemented with nuts; LFD, low-fat diet; MUFA, Monounsaturated fatty acids; PUFA, Polyunsaturated fatty acids; Refined OO, refined olive oil; SFA, Saturated fatty acids.
